# Incentivized walking improves chronic disease indicators: a short-term field intervention among an occupational population in Southeast China

**DOI:** 10.3389/fpubh.2026.1746055

**Published:** 2026-01-27

**Authors:** Xiangju Hu, Menglin Yu, Zhifeng Lin, Minxia Wu, Zhijian Hu

**Affiliations:** 1 Fujian Provincial Key Laboratory of Environment Factors and Cancer, Department of Epidemiology and Health Statistics, School of Public Health, Fujian Medical University, Fuzhou, China; 2 Department for Chronic and Noncommunicable Disease Control and Prevention, Fujian Provincial Center for Disease Control and Prevention, Fuzhou, Fujian, China; 3 Public Technology Service Center, Fujian Medical University, Fuzhou, China; 4 Department of Key Laboratory of the Ministry of Education for Gastrointestinal Cancer, Fujian Medical University, Fuzhou, China

**Keywords:** chronic disease, health indicators, motivational mechanism, professional groups, scientific walking

## Abstract

**Purpose:**

This study aimed to evaluate the effect of a short-term incentivized walking intervention on chronic disease indicators in an occupational cohort, thereby exploring effective strategies for chronic disease prevention and control.

**Methods:**

In a 100-day supervised walking intervention, a cohort of occupational participants was motivated by an individual- and team-based incentive system, with step counts tracked by a uniform pedometer. Key anthropometric measures (height, weight, waist and hip circumference, waist-to-hip ratio, and body fat percentage) were collected at baseline and the study endpoint. Data analysis was performed using SPSS 26.0.

**Results:**

All participants successfully completed the 100-day scientific walking intervention, with 936 (64.2%) achieving perfect adherence to the daily 10,000-step goal. The final assessment showed that all eight chronic disease-related indicators (systolic blood pressure, diastolic blood pressure, body weight, BMI, waist circumference, hip circumference, waist-to-hip ratio, and body fat percentage) improved in both male and female participants., with reductions in systolic blood pressure (2.47 mmHg), diastolic blood pressure (4.23 mmHg), body weight (1.60 kg), BMI (0.57 kg/m^2^), waist circumference (1.33 cm), hip circumference (0.86 cm), waist-to-hip ratio (0.01), and body fat percentage (0.01%). Furthermore, a dose-dependent relationship was observed, wherein greater walking intensity led to more pronounced improvements across all metrics.

**Conclusion:**

The short-term scientific walking intervention was associated with significant improvements in multiple physical indicators, including systolic and diastolic blood pressure, waist circumference, waist-to-hip ratio, BMI, and body fat percentage. Reductions in BMI and waist circumference were particularly notable. These findings may help inform workplace health strategies for reducing non-communicable disease (NCD) risk, though further controlled studies are necessary to establish causal relationships.

## Introduction

Chronic non-communicable diseases (NCDs) have emerged as a major public health challenge globally. According to the World Health Organization (WHO), physical inactivity represents the fourth leading risk factor for mortality worldwide (1). In China, however, the level of participation in physical activity remains low. Among residents aged 18 and above, only 18.7% engage in regular exercise, and this proportion drops to just 9.9% among the working-age population aged 25–34 (2). This widespread lack of adequate physical activity has contributed to rising rates of morbidity and mortality from NCDs such as hypertension, diabetes, and cardiovascular diseases (3).

Scientific walking presents a safe, accessible, and sustainable form of exercise suitable for a broad population. In this study, scientific walking refers to a cadence-defined brisk-walking prescription (100–150 steps/min) performed for more than 35 min per day, with ‘effective steps’ captured by a standardized pedometer to ensure moderate-intensity activity. Evidence shows that it can help reduce body weight, waist circumference, and body fat in individuals with obesity, while also improving overall health and quality of life—particularly among middle-aged and older adults—and enhancing psychological well-being. Given its convenience and flexibility, scientific walking is especially well-suited for the prevention and management of NCDs in occupational groups, as it can be easily integrated into daily routines without being constrained by time or space (4).

The NIOSH Total Worker Health^®^ (TWH) framework offers a comprehensive approach to integrating occupational safety and health protection with workplace health promotion (5). This model recognizes the interconnection between work-related factors and personal health, advocating for interventions that simultaneously address the work environment and individual behaviors (6). Scientific walking aligns closely with TWH principles by providing a practical, low-cost strategy that can be readily incorporated into the workplace to encourage physical activity and enhance overall well-being.

Scientific walking originated in Europe and has since been widely promoted across numerous Western countries. A U.S. study involving 21 sedentary adults aged 40–64 with dyslipidemia found that a 12-week walking intervention (30–60 min daily, 10,000 steps/day) significantly improved high-density lipoprotein cholesterol, although it also led to increases in low-density lipoprotein and total cholesterol levels (7). Similarly, a Japanese study of older patients with cardiovascular disease during the maintenance phase of cardiac rehabilitation showed that both community-based and individually managed walking groups significantly increased physical activity over 3 months, with the community group also exhibiting more positive trends in subjective well-being (8).

In China, the empirical focus of studies on scientific walking has largely been on its influence on physical health parameters, including weight, blood pressure, blood glucose, BMI, body fat percentage, and visceral fat (9). Meanwhile, research outside China commonly employs a broader set of endpoints that encompass quality-of-life dimensions such as sleep quality, psychological well-being, and the capacity to perform daily activities.

There remains a scarcity of research on the application of scientific walking interventions in China. To address this gap, this study implemented a 100-day scientific walking intervention among a sedentary occupational population in Fujian, a southeastern coastal province of China. The intervention aimed to promote the adoption of scientific walking, investigate the formation and dissemination patterns of healthy behaviors and lifestyles, and evaluate its effects on preventing and managing common NCDs. Ultimately, this research seeks to establish a sustainable model for NCD prevention within occupational groups.

## Methods

### Study population

The study enrolled 1,458 participants aged 18 to 60 years from 68 organizations across Fujian Province, China. The organizations included state agencies, public institutions, state-owned enterprises, and private enterprises, all located within 12 Demonstration Zones of Chronic Disease Prevention and Control (DZCDC) spanning nine cities. Each participating unit was required to recruit at least 20 participants or at least 80% of its total employees. Participants engaged in scientific walking through both team-based and individual formats. Teams consisted of 10 to 20 members, each led by a designated team leader responsible for supervision and management. During the intervention, participants who did not meet the activity requirements received timely reminders and supervision from their team leaders.

### Inclusion criteria

Participants were required to meet all of the following criteria:

1. Employed as staff at a government agency, mass organization, enterprise, or public institution, and aged 18 years or older.
2. Reported a sedentary or irregularly active lifestyle prior to the study, defined as engaging in moderate-intensity exercise fewer than 3 times per week, with each session lasting less than 30 min.
3. Provided informed consent.
4. Had no severe chronic diseases (e.g., hypertension, asthma, severe cardiac or cerebrovascular conditions) or other physical or mental contraindications (e.g., major depression or other serious mental disorders) that would preclude safe participation in a walking intervention.

### Exclusion criteria

Participants were excluded based on the following criteria:

(1) Inability to use a smart phone proficiently.
(2) Unwillingness or inability to participate in brisk walking exercises or to complete the required physical examinations.
(3) Unwillingness to cooperate in completing the questionnaire survey.

### Questionnaires and physical examinations

Standardized face-to-face questionnaires and physical examinations were conducted at baseline and upon completion of the 100-day scientific walking intervention. The questionnaire covered demographic characteristics (e.g., gender, age, marital status, education, occupation), medical and family history, smoking status, dietary habits, physical activity, sleep quality, and psychological status. Physical examinations included measurements of height, weight, waist circumference, hip circumference, blood pressure, body fat percentage, and visceral fat index.

Specific instruments and protocols were employed as follows: a BCA-1C body composition analyzer (Tongfang Health Technology, Beijing) was utilized to measure body weight, body fat percentage, and visceral fat index. Measurements for height and waist circumference were obtained using a stadiometer and waist-hip calipers, respectively, adhering to the procedures stipulated in the Chinese health industry standard “Anthropometric Methods for Population Health Surveillance (WS/T424-2013),” with a measurement precision of 0.1 cm.

All data collectors were uniformly trained and certified to ensure consistent application of methods and use of instruments across all study sites. Prior to inclusion, written informed consent was secured from every participant, affirming their voluntary involvement. The study received ethical approval from the Ethics Committee of the Center for Chronic Non-communicable Disease Control and Prevention, Chinese Center for Disease Control and Prevention (Approval No. 201607).

### Intervention of scientific walking

Participants engaged in a scientific walking program from May 11 to August 18. Following standardized training led by a professional coach, they wore a pedometer (Model: TW736, Beijing Wanbu Health Technology Co., Ltd.) to track daily step counts, step frequency, stride length, and duration. All recorded data were subsequently uploaded to the Wanbu system.

### Incentive scores approach of scientific walking

An incentive scoring system was established to promote both individual and team motivation, based on a daily step goal of 10,000. The daily scoring rules were as follows: a baseline of 4 points was awarded for reaching 6,000 steps. Beyond this, 1 additional point was granted for every further 1,000 steps accumulated, culminating in a maximum of 8 points for achieving the 10,000-step target. Furthermore, to ensure steps were taken at a moderate exercise intensity, the pedometer was set to record only “effective steps”-defined as those taken at a cadence of 100–150 steps per minute. The 10,000-step target refers to ‘effective steps’ accumulated at 100–150 steps/min.

### Participatory motivational activities

In addition to the calculation of walking scores, participatory motivational activities were implemented by each DZCDC. Points earned from two selected activities contributed to the team’s overall incentive score. These activities were categorized into three distinct types:

(1) Team Activities, which encompassed thematic walking weeks, field walking sessions, training for step leaders, discovery of walking routes, health lectures, and a launch ceremony.
(2) Individual Activities, which included an essay contest, questionnaires, collection of health indicators, brisk walking training, and a photography contest.
(3) Independent Incentive Mechanisms, defined as locally innovated incentives that could involve material/spiritual rewards, integration into work assessments, or funding support policies.

The corresponding scoring rules are presented in Table 1.

**Table 1:** Scoring rules of the scientific walking incentive intervention for the professional population.

Team activities	Incentive events	Score
Launch ceremony	More than 80% of participants on site	3
	Group photo, warm up, stretching, and walking more than 2 photos respectively	3
	Leadership participation: Section level Division Division level above	3 6 10
	Wanbu special column releases launch ceremony report	2
Thematic walking week activities	Group photo, warm up, and walking more than 5 photos respectively	4
	Creative performance 5 or more photos	5
	Game session: 3 games 2 games 1 game	7 4 1
	Have obvious field walking signs and more than 5 publicity boards	10
	Wanbu special column releases field walking activity report	3
Field walking	More than 30% of participants on site	1
	Group photo, warm up, stretching, and walking more than 2 photos respectively	1
	Have 1–2 leaders participate	2
	Wanbu special column releases field walking activity report	1
	Have obvious field walking signs and more than 2 publicity boards	2
10,000 steps leader training	For each leader trained, awarded points to the demonstration area, with a full of 50 scores.	5
Discovery of walking sites	For each walking site found, awarded points to the demonstration area, with a full of 50 scores.	5
Health lectures	More than 30 participants signed up on site	2
	More than 5 photos including group photo, interaction, detail demonstration, action demonstration	2
Individual activities essay contest	First prize (top 5%)	30
	Second prize (top 6–10%)	20
	Third prize (top 11%~15%)	15
	Finalists (top 16–20%)	10
Photography contest	First prize (top 5%)	30
	Second prize (top 6–10%)	20
	Third prize (top 11%~15%)	15
	Finalists (top 16–20%)	10
Brisk walking training	The correct rate of examination is 80% or more	10
Questionnaire and health indicators collection	Participate the pre-intervention questionnaire only	3
	Participate the post-intervention questionnaire only	3
	Participate the pre- and post-intervention questionnaire	10
	Participate in the pre-intervention physical test only	3
	Participate in the post-intervention physical test only	3
	Participate in the pre-and post-intervention physical test	10
Independent incentive mechanism	Setting commendation and reward method	6
	Establishing the system of walking,	5
	Other innovative incentives	5
	Complete textual information	2
	Clear and complete picture information	2

### Indicators and definition

1. Scientific Walking: Defined as a brisk walking regimen with prescribed cadence, to be performed for at least 35 min daily. The target cadence is maintained between 100 and 150 steps per minute, with “effective steps” monitored via a standardized pedometer to ensure moderate-intensity physical activity (10).
2. 10,000 Steps Rate: This metric refers to the proportion of the total intervention days (100 days) on which a participant’s daily effective step count reached or exceeded 10,000 steps. It was calculated as follows: (Number of days with ≥10,000 steps / 100 days). Both the 10,000-step goal and incentive scoring were based on steps accumulated within the cadence-defined “scientific walking” range (100–150 steps/min), as captured by the pedometer/app. Steps recorded outside this cadence range were logged by the device but were not counted toward the “scientific walking” step target for scoring purposes (11).
3. Exercise Prescription: The prescribed walking regimen required participants to walk at a cadence of 100–150 steps per minute, accumulating at least 35 min per day. The activity could be completed in multiple bouts (e.g., 10, 10, and 15 min) and had to be performed between 05:00 and 23:00. With a minimum weekly total of approximately 245 min, this prescription exceeds the WHO recommendation of 150 min of moderate-intensity physical activity per week for adults. This relatively high weekly volume may help explain the short-term changes observed in the study, though the long-term sustainability of such a regimen warrants further investigation.
4. Prescription Completion Rate: Defined as the number of days on which the exercise prescription was fully completed, divided by the total number of days in the intervention period (100 days).
5. Daily Average Steps: The average number of steps taken per day by a participant throughout the 100-day intervention, derived from the cumulative step count divided by 100.
6. Central Obesity: Classified using waist circumference thresholds, defined as ≥90 cm for males and ≥85 cm for females (12).
7. Body Mass Index (BMI): A common metric for assessing weight status, calculated as weight (kg) divided by the square of height (m^2^). Classification is defined as follows: <18.5 (underweight), 18.5–23.9 (normal weight), 24.0–27.9 (overweight), and ≥28.0 (obese) (13).
8. Body Fat Percentage (BFP): The ratio of adipose tissue mass to total body mass, expressing the relative fat content in the body.
9. Waist-to-Hip Ratio (WHR): An index of body fat distribution, calculated as the circumference of the waist (cm) divided by the circumference of the hips (cm).

### Statistical methods

Data were double-entered using EpiData 3.1 and analyzed with SPSS 26.0. Normality was assessed using Q-Q plots/histograms and the Shapiro–Wilk test. Parameters including age, weight, body mass index, systolic and diastolic blood pressure, waist circumference, hip circumference, and waist-to-hip ratio approximate a normal distribution are summarized as mean±standard deviation ( $\bar{x}$ ±s). Non-normally distributed variables-specifically prescription fulfillment rate and 10,000-step achievement rate-are reported as median values with interquartile ranges (Q25, Q75). Categorical data are expressed as frequencies (*N*) and percentages (%). Group comparisons were conducted using appropriate statistical tests based on data distribution and variable type:

1. Independent samples *t*-tests and one-way ANOVA were employed to compare mean daily step counts across demographic subgroups.
2. Mann–Whitney(Z) and Kruskal-Wallis(H) tests were used to analyze differences in 10,000-step rates and prescription fulfillment rates across participant characteristics.
3. Paired *t*-tests evaluated pre- and post-intervention changes in physical health indicators.
4. One-way ANOVA examined variation in health indicator improvements across different 10,000-step achievement groups.
5. Multivariable logistic regression identified factors associated with consistent achievement of daily 10,000-step goals.

All statistical tests were two-tailed, with significance defined as *p* < 0.05. Complete variable definitions and coding schemes are provided in Table 2.

**Table 2:** Assignment value of variables for multifactorial logistic regression analysis.

Variables	Assignment value
Age (Year)	1 = <35, 2 = 35–, 3 = 50–
Gender	1 = Male, 2 = Female
Occupation	1 = Leader, 2 = Professionals, 3 = Clerical personnel, 4 = others
Marriage	1 = Unmarried, 2 = Married, 3 = Divorced
Company type	1 = Government agency, 2 = Institution, 3 = State-owned enterprises, 4 = Private enterprises, 5 = others
Education	1 = Elementary School, 2 = Middle School, 3 = High School, 4 = College or above
Prescription completion rate = 100%	0 = No, 1 = Yes
Average daily steps ≥10,000	0 = No, 1 = Yes
Hypertension	0 = No, 1 = Yes
Overweight and obesity	0 = No, 1 = Yes
Central obesity	0 = No, 1 = Yes
10,000 steps rate = 100%	0 = No, 1 = Yes

## Result

### Characteristics of the participants

Following data diagnosis and cleaning for logical errors, outliers, and missing values, 1,458 participants were included in the analysis, containing a higher proportion of females (54.9%, 800/1,458) than males (45.1%, 658/1,458), with an average age of (39.07 ± 9.77) years. The majority of participants were aged 18–35 years (34.4%, 502/1,458), while the smallest proportion belonged to the 55–60 age group (5.6%, 81/1,458). Most participants were married (82.0%, 1,195/1,458) and possessed a high education level or above (98.1%, 1,431/1,458), compared to only 1.9% with middle school education or below. In terms of occupational background, the vast majority (89.9%, 1,311/1,458) were from state agencies and institutions. Regarding occupational roles, leaders of agencies accounted for 22.4% (*n* = 326), professional and technical personnel for 53.9% (*n* = 787), and other clerical staff for 23.7% (*n* = 345).

The prevalence of overweight and obesity, hypertension, and central obesity was 43.55% (635/1,458), 9.60% (140/1,458), and 19.50% (284/1,458), respectively. Mean values for body composition were as follows: body fat percentage, 23.91 ± 10.82%; waist circumference, 80.69 ± 9.06 cm; hip circumference, 93.80 ± 8.12 cm; and waist-hip ratio, 0.86 ± 0.10.

Regarding intervention metrics, the median 10,000 steps rate was 100% (IQR:97, 100), the average daily steps were 1.39 ± 0.32 (in tens of thousands, presumably), and the median prescription fulfillment rate was 100% (IQR:93, 100). The baseline characteristics of the participants are presented in Table 3.

**Table 3:** Baseline characteristics of study population.

Variables	*N*	$\bar{x}$ *±s* (Q25, Q75)	Ratio (%)	Composition ratio (%)
Gender				
Male	658	–	–	45.13
Female	800	–	–	54.87
Age (Year)		39.07 ± 9.77		
<35	502	–	–	34.43
35-	735	–	–	50.41
≥50	221	–	–	15.16
Education				
College or above	1,431	–	–	98.15
High school	17	–	–	1.16
Middle school	7	–	–	0.48
Elementary school	3	–	–	0.21
Marriage				
Unmarried	250	–	–	17.15
Married	1,190	–	–	81.62
Divorced	18	–	–	1.23
Company type				
Government agency	265	–	–	18.18
Institution	1,003	–	–	68.79
State-owned enterprises	101	–	–	6.93
Private enterprises	46	–	–	3.16
Others	43	–	–	2.94
Occupation				
Leader	326	–	–	22.36
Professionals	715	–	–	49.04
Clerical personnel	312	–	–	21.40
Others	105	–	–	7.20
Overweight and obesity	635	–	43.55	–
Central obesity	284	–	19.50	–
Hypertension	140	–	9.60	–
Systolic blood pressure	–	119.42 ± 15.39	–	–
Diastolic blood pressure	–	79.92 ± 11.60	–	–
BMI	–	23.66 ± 3.23	–	–
Waist circumference(cm)	–	80.69 ± 9.06	–	–
Hip circumference(cm)	–	93.80 ± 8.12	–	–
Waist-hip ratio	–	0.86 ± 0.10	–	–
Prescription fulfillment rate (%)	–	100(93,100)	–	–
10,000 steps rate (%)	–	100(97,100)	–	–
Average daily steps (10,000)	–	1.39 ± 0.32	–	–

### Implementation of scientific walking

The assessment of participants’ adherence to scientifically-guided walking was based on three indicators: average daily steps, 10,000 steps rate, and prescription completion rate. The results indicated that the average daily steps exceeded 10,000, at the same time, both the median 10,000 steps rate and prescription completion rate reached 100%. Men demonstrated significantly higher average daily steps than women (*p* < 0.05). Furthermore, all three indicators were relatively higher among specific subgroups, including older adults (≥50 years), clerical staff, individuals with central obesity, and those with a college education or above, with all differences being statistically significant (*p* < 0.05). The implementation of scientific walking for the different characteristic population was presented in Table 4. The high adherence rates observed in this study (e.g., 85% achieving the 10,000-step target) may be partially attributable to the incentive structure and team-based supervision framework, which may not be sustainable in routine workplace settings without such support systems. While these strategies were effective in promoting engagement during the intervention period, their long-term feasibility and cost-effectiveness in real-world implementation require further evaluation.

**Table 4:** Completion of scientific walking activities by study population.

Characteristics of population		N	Average daily step (10,000)	10,000 step rate^*^(%)	Prescription completion rates^*^(%)
Gender	Male	658	1.43 ± 0.35	100(96,100)	100(92,100)
	Female	800	1.36 ± 0.30	100(97,100)	100(94,100)
	*t*(*Z*)		3.85	−0.67	−0.49
	*P*		<0.01	>0.05	>0.05
Age (Year)	<35	502	1.34 ± 0.30	100(94,100)	100(91,100)
	35-	735	1.39 ± 0.31	100(97,100)	100(94,100)
	≥50	221	1.49 ± 0.39	100(99,100)	100(97,100)
	*F*(*H*)		16.88	16.54	8.44
	*P*		<0.01	<0.05	<0.05
Occupation	Leader	326	1.37 ± 0.39	99(90,100)	97(84,100)
	professionals	715	1.39 ± 0.31	100(98,100)	100(94,100)
	Clerical personnel	312	1.41 ± 0.28	100(100,100)	100(99,100)
	others	105	1.42 ± 0.33	100(100,100)	100(100,100)
	*F*(*H*)		1.03	89.76	92.6
	*P*		>0.05	<0.01	<0.01
Education	College or above	1,431	1.39 ± 0.33	100(97,100)	100(94,100)
	High school or below	27	1.29 ± 0.18	100(80,100)	96(73,100)
	*t*(*Z*)		2.87	−2.12	−2.81
	*P*		<0.01	<0.05	<0.01
Central obesity	Yes	284	1.42 ± 0.28	100(100,100)	100(99,100)
	No	1,174	1.38 ± 0.33	100(95,100)	100(92,100)
	*t*(*Z*)		2.08	−5.48	−6.10
	*P*		<0.05	<0.01	<0.01

^*^Average daily steps are expressed as x ± s, and 10,000 step rates, and prescription completion rates are expressed as medians (Q25, Q75).

### Comparison of physical indicators in participants pre- and post-intervention

After 100 days of the scientific walking intervention, all measured physiological indicators—including SBP, DBP, body weight, BMI, waist circumference, hip circumference, and body fat percentage—showed significant decreases (*p* < 0.01) in both male and female participants, with the exception of the waist-to-hip ratio. Detailed pre- and post-intervention data for these health indicators are presented in Table 5.

**Table 5:** Comparison of physical indicators before and after scientific walking of different genders.

Variables	Male			Female			Total		
	Before intervention	After the intervention	*P*	Before intervention	After the intervention	*P*	Before intervention	After the intervention	*P*
SBP	122.63 ± 16.26	119.97 ± 13.46	<0.01	116.79 ± 14.11	114.47 ± 10.56	<0.01	119.42 ± 15.39	116.95 ± 12.26	<0.01
DBP	81.73 ± 11.82	77.33 ± 9.75	<0.01	78.43 ± 11.21	74.34 ± 9.44	<0.01	79.92 ± 11.60	75.69 ± 9.69	<0.01
Weight (kg)	72.75 ± 10.06	70.84 ± 9.81	<0.01	58.69 ± 8.46	57.35 ± 8.09	<0.01	65.04 ± 11.57	63.44 ± 11.15	<0.01
BMI	24.73 ± 3.14	24.06 ± 3.01	<0.01	22.79 ± 3.03	22.3 ± 2.91	<0.01	23.66 ± 3.23	23.09 ± 3.08	<0.01
Waist (cm)	84.72 ± 8.21	83.25 ± 7.80	<0.01	77.38 ± 8.36	76.16 ± 7.94	<0.01	80.69 ± 9.06	79.36 ± 8.63	<0.01
Hip circumference (cm)	95.19 ± 7.87	94.30 ± 6.88	<0.01	92.66 ± 8.15	91.83 ± 7.21	<0.01	93.80 ± 8.12	92.95 ± 7.17	<0.01
Waist-to-hip ratio	0.89 ± 0.09	0.88 ± 0.06	<0.01	0.84 ± 0.10	0.83 ± 0.06	<0.01	0.88 ± 0.08	0.85 ± 0.07	>0.05
BFP (%)	16.42 ± 7.15	15.63 ± 7.03	<0.01	30.07 ± 9.34	29.37 ± 9.26	<0.01	23.91 ± 10.82	23.17 ± 10.77	<0.01

### Effect of scientific walking on the reduction of physical indicators

During the 100-day walking intervention, participants showed reductions in SBP, DBP, body weight, BMI, waist circumference, hip circumference, waist-to-hip ratio, and body fat percentage by 2.47 mmHg, 4.23 mmHg, 1.60 kg, 0.57 kg/m^2^, 1.33 cm, 0.86 cm, 0.01, and 0.01%, respectively. More importantly, the magnitude of these improvements demonstrated a clear dose–response relationship with implementation intensity, with higher adherence levels associated with greater improvements. Participants were categorized by three implementation intensity levels based on the 10,000 steps rate: <80, 80–90, and ≥90%. Significant differences in pre- and post-intervention changes were observed in SBP, body weight, BMI, and waist circumference across these groups (*p* < 0.05), while changes in DBP, hip circumference, waist-to-hip ratio, and body fat percentage did not differ significantly (*p* > 0.05).

When grouped by average daily steps (<10,000 vs. ≥10,000 steps), significant differences in changes were found for SBP, body weight, BMI, waist circumference, and waist-to-hip ratio (*p* < 0.05), while not for DBP, hip circumference, or body fat percentage (*p* > 0.05). Similarly, based on prescription completion rate (<100% vs. 100%), significant between-group differences were observed in SBP, DBP, and body weight (*p* < 0.05), whereas no significant differences were detected in waist circumference, hip circumference, waist-to-hip ratio, or body fat percentage (*p* > 0.05). The relationship between the decline in physical indicators and the implementation of scientific walking is detailed in Table 6.

**Table 6:** Comparison of the decline in physical indicators with the implementation of scientific walking.

Decline in physical indicators^*^	10,000 steps rate (%)				Average daily steps				Prescription completion rate (%)			
	≥90 (*N* = 1,242)	80–90 (*N* = 82)	<80 (*N* = 134)	*P*	<10,000 (*N* = 71)	≥10,000 (*N* = 1,387)	*P*	Mean change 95% CI	<100 (*N* = 621)	100 (*N* = 837)	*P*	Mean change 95% CI
SBP (mmHg)	3.13 ± 17.51	−0.05 ± 11.45	−2.14 ± 7.81	<0.01	−0.50 ± 8.86	2.62 ± 16.92	<0.01	(−5.40 to −0.85)	−2.00 ± 10.86	5.79 ± 19.20	<0.01	(−9.35 to −6.24)
DBP (mmHg)	4.21 ± 14.04	4.03 ± 8.20	4.53 ± 5.93	>0.05	5.18 ± 3.83	4.18 ± 13.53	>0.05	(−0.15 to 2.14)	3.33 ± 9.29	4.90 ± 15.49	<0.05	(−2.86 to −0.30)
Weight (kg)	1.7 ± 4.06	0.55 ± 6.46	1.24 ± 2.79	<0.05	0.87 ± 2.49	1.63 ± 4.21	<0.05	(−1.39 to −1.32)	0.86 ± 3.71	2.14 ± 4.36	<0.01	(−1.70 to −0.87)
BMI (kg/m^2^)	0.61 ± 1.50	0.17 ± 2.30	0.43 ± 0.97	<0.05	0.31 ± 0.92	0.58 ± 1.54	<0.05	(−0.50 to −0.04)	0.29 ± 1.35	0.78 ± 1.60	<0.01	(−0.64 to −0.33)
WC (cm)	1.43 ± 4.08	1.22 ± 4.04	0.49 ± 3.43	<0.05	0.48 ± 3.29	1.38 ± 4.05	<0.05	(−1.71 to −0.09)	1.25 ± 4.37	1.39 ± 3.74	>0.05	(−0.56 to 0.29)
HC (cm)	0.84 ± 3.95	1.13 ± 3.82	0.85 ± 1.74	>0.05	0.97 ± 1.62	0.85 ± 3.86	>0.05	(−0.78 to 1.03)	0.93 ± 4.06	0.81 ± 3.57	>0.05	(−0.27 to 0.51)
WHR	0.01 ± 0.08	0.00 ± 0.03	0.00 ± 0.03	>0.05	0.00 ± 0.04	0.01 ± 0.09	<0.01	(−0.022 to −0.003)	0.01 ± 0.09	0.01 ± 0.07	>0.05	(−0.011 to 0.005)
BFP (%)	0.01 ± 0.05	0.01 ± 0.05	0.00 ± 0.04	>0.05	0.00 ± 0.05	0.01 ± 0.05	>0.05	(−0.02 to 0.01)	0.01 ± 0.05	0.01 ± 0.05	>0.05	(−0.001 to 0.009)

SBP, systolic blood pressure; DBP, diastolic blood pressure; BMI, body mass index; WC, waist circumference; HC, hip circumference; WHR, waist-to-hip ratio; BFP, body fat percentage.

### Multifactorial logistic regression analysis of 10,000 step rate completion

A multifactor unconditional logistic regression analysis was conducted to identify factors associated with the 10,000-step completion rate. The completion rate of 10,000-step set as the dependent variable, and independent variables included gender, marital status, age, employment status, occupation, education level, hypertension, overweight and obesity, central obesity, and daily average steps. The results indicated that older age, being clerical staff, hypertension, central obesity, prescription completion rate, and higher daily average steps were significant influencing factors (*p* < 0.05), as detailed in Table 7.

**Table 7:** Multifactorial logistic regression analysis for the completion of the ten-thousand-step rate.

Factors		β	S.E	Waldχ^2^	OR(95%CI)	*P*
Gender	Male	1.00				
	Female	0.20	0.24	0.71	1.23(0.76–1.97)	>0.05
Age (Year)	<35	1.00				
	35–	0.91	0.4	5.05	2.47(1.12–5.45)	<0.01
	≥50	2.48	0.68	13.08	11.93(3.11–45.71)	<0.01
Company type	Government agency	1.00				
	Institution	−0.25	0.31	0.66	0.78(0.42–1.44)	>0.05
	State-owned enterprises	−0.37	0.48	0.60	0.69(0.27–1.76)	>0.05
	Private enterprises	0.46	0.65	0.50	1.59(0.44–5.69)	>0.05
	Others	1.06	0.63	2.80	2.87(0.83–9.88)	>0.05
Occupation	Leader	1.00				
	professionals	0.40	0.27	2.16	1.49(0.87–2.54)	>0.05
	Clerical personnel	1.01	0.33	9.34	2.73(1.43–5.21)	<0.01
	Others	1.06	0.57	3.46	2.87(0.94–8.76)	>0.05
Education	College or above	1.00				
	High school	−0.93	0.80	1.37	0.39(0.08–1.87)	>0.05
	Middle school	2.54	1.38	3.40	12.66(0.85–187.90)	>0.05
	Elementary school	−2.20	3.41	0.42	0.11(0.00–87.81)	>0.05
Marriage	Unmarried	1.00				
	Married	−0.55	0.30	3.43	0.57(0.32–1.03)	>0.05
	Divorced	−0.55	1.11	0.25	0.57(0.06–5.02)	>0.05
	Others	−0.37	1.33	0.08	0.69(0.05–9.29)	>0.05
Hypertension		1.71	0.72	5.58	5.52(1.34–22.77)	<0.05
Overweight and obesity		−0.48	0.19	6.39	0.62(0.43–0.90)	<0.05
Central obesity		2.18	1.09	3.97	8.84(1.04–75.33)	<0.05
Prescription completion rate		5.61	0.42	180.72	274.11(120.91–621.41)	<0.01
Average daily steps		6.31	0.731	74.54	549.72(131.25–2302.44)	<0.01

## Discussion

In recent decades, accelerated urbanization in China has led to significant shifts in residents’ transportation habits and lifestyles, particularly among professional groups. These individuals often adopt sedentary routines with insufficient physical activity, posing considerable risks to their health. According to the “Global Burden of Disease” report, 21 million premature deaths worldwide in 2023 were attributed to low levels of physical activity (14). In response, the Scientific Walking Activity was launched as a chronic disease intervention initiative organized by the Center for Chronic and Non-communicable Disease Control and Prevention at the China Center for Disease Control and Prevention. Utilizing exercise prescription pedometers and “Internet + health” technologies, this program tracks participants’ daily step counts, walking duration, and frequency. By leveraging the supervisory and motivational framework of “teams” formed within government agencies, public institutions, state-owned enterprises, and private companies, the project aims to help participants cultivate a healthy habit of walking 10,000 steps per day, thereby preventing chronic diseases and promoting overall health.

Participant adherence is a critical factor for the success of intervention studies. Extensive research has established that employing varied incentive structures is one of the most effective strategies to improve compliance. In the present study, we implemented a comprehensive set of team and individual incentives. Team incentives included a demonstration area launch ceremony, thematic walking weeks, organized field walking, the nomination of “10,000 Steps Leaders,” the discovery of walking sites, and health lectures. Individual incentives comprised an essay contest, health indicator collection, walking training, and a photography contest. This multi-faceted approach significantly elevated participants’ awareness and enthusiasm (15). The incentive mechanism facilitated the development of a scientific walking habit among participants, effectively enabling a high rate of adherence to the 10,000-step daily target. Specifically, 936 of the 1,458 participants (64.2%) successfully achieved 10,000 steps per day for 100 consecutive days. Our analysis indicates that team-based participatory activities and self-motivation mechanisms were effective in promoting daily and concentrated walking practice. Conversely, individual participatory activities were beneficial in enhancing the number of steps, stride length, and frequency of scientific walking. These observations are consistent with the findings of previous studies, which demonstrate that establishing outdoor walking teams can promote and maintain scientific walking behavior at the group level (16, 17). The insights garnered from this study will be of positive significance for informing and improving the design of future scientific walking interventions for the occupational population in China.

A pre-post analysis of the short-term brisk walking intervention demonstrated consistent improvements across all physical health indicators measured. Specifically, reductions were observed in systolic and diastolic blood pressure, body weight, waist circumference, hip circumference, waist-to-hip ratio, BMI, and body fat percentage. These findings align with previous research (18, 19) and indicate that even a short-term, continuous regimen of scientifically-guided walking can effectively improve chronic disease-related physiological parameters in occupational populations. While improvements were seen across all intervention groups, the extent of reduction in physical indicators varied, with greater reductions consistently associated with higher adherence to the prescribed walking intensity. It should be noted that the current study focused mainly on anthropometric and physiological outcomes. This focus reflects the broader research landscape of scientific walking in China, which has traditionally prioritized physical health measures (20). In contrast, international studies often include a wider range of endpoints—such as sleep quality, psychological well-being, and health-related quality of life—which were not evaluated in the present study (21).

A multifactorial logistic regression analysis was conducted to identify factors associated with the 10,000-step rate. Results indicated that an educational background of high school or below, as well as employment in a state-owned enterprise, were both significantly associated with a lower 10,000-step rate (*p* < 0.05). These findings align with existing literature reporting a positive relationship between educational level and the frequency, consistency, and scientific approach to physical activity (22, 23). Individuals with lower educational attainment may have a relatively limited understanding of the health benefits associated with regular physical activity, such as walking, leading to lower motivation to proactively engage in walking for health promotion (24). Furthermore, many positions in state-owned enterprises involve administrative, clerical, or production-monitoring duties, which are predominantly sedentary and thus offer limited occupational physical activity (25).

In contrast, older age and being technical personnel were significantly associated with a higher step rate (*p* < 0.05). This is consistent with studies suggesting that older adults often show stronger motivation for physical activity, as they tend to have clearer fitness goals and better recognize how regular exercise contributes to physical health, immune function, and longevity (26, 27). Additionally, compared to administrative roles or shift-based jobs, technical positions generally provide greater job autonomy and more flexible schedules, which can facilitate the integration of regular walking into daily routines (28).

Although the observed improvements in physiological indicators were statistically significant, their clinical relevance requires careful interpretation. For example, the mean reduction in systolic blood pressure of 2.47 mmHg, while statistically significant, may fall short of the threshold generally regarded as clinically meaningful (typically defined as a reduction of ≥5 mmHg in hypertension management). Similarly, the weight reduction observed, though statistically detectable, may represent only modest improvement with limited short-term clinical impact. However, it is noteworthy that even small, sustained improvements across multiple cardiovascular risk factors can collectively contribute to meaningful long-term health benefits, especially in primary prevention contexts. Future studies with extended follow-up periods are necessary to determine whether these modest changes are sustained and whether they translate into reduced incidence of cardiovascular events or other clinically significant endpoints.

Regarding the non-significant changes observed in certain indicators, several physiological and methodological factors may account for these findings. First, diastolic blood pressure (DBP) is generally less responsive than systolic blood pressure to short-term, moderate-intensity physical activity interventions, as it is governed by distinct hemodynamic mechanisms and may require longer or more intensive interventions to produce measurable changes (29, 30). Second, hip circumference and waist-to-hip ratio (WHR) are composite measures that tend to be relatively insensitive to modest, short-term changes in body composition, especially when alterations in fat distribution are subtle or localized (31). Finally, the lack of significant change in body fat percentage (BFP) may reflect the fact that substantial reductions in body fat typically require interventions of longer duration, higher intensity, or combined dietary modifications (32, 33). These observations underscore the importance of considering intervention duration, intensity, and outcome selection when assessing the effectiveness of lifestyle interventions in similar populations.

The “Healthy China 2030” Outline emphasizes the need to develop and implement physical health intervention programs for occupational groups and other specific populations (34). In alignment with this objective, the Medium- and Long-Term Plan for the Prevention and Control of Chronic Diseases in China (2017–2025) encourages public institutions, enterprises, and other organizations to promote activities such as workplace fitness programs, staff sports competitions, healthy walking, and health knowledge contests (35). In accordance with the national policy of deepening healthcare reform and advancing physical fitness initiatives, this study implemented a brisk walking intervention among occupational groups in southeastern China. By incorporating the “Internet + health” model, the study contributes to the practical advancement of the “Healthy China” initiative and fosters a health-conscious lifestyle among working individuals (36–38). Furthermore, it offers valuable insights and technical support for developing effective intervention strategies tailored to chronic disease prevention and control in China’s occupational population.

## Limitations

This study has several limitations. First, the voluntary, self-selected nature of enrollment may have introduced selection bias, as participants were likely more motivated than the general working population, potentially inflating adherence and limiting the sensitivity of subgroup comparisons. Second, the sample consisted predominantly of public-sector office workers, which may restrict the generalizability of findings to blue-collar, shift-work, or more physically active occupational groups. Third, the absence of a parallel control group prevents causal inference, as observed improvements may reflect secular trends, regression to the mean, Hawthorne effects, or unmeasured co-interventions. Fourth, the outcomes were confined to physiological indicators; patient-centered outcomes such as sleep quality, mental well-being, and health-related quality of life were not assessed. Finally, the 100-day intervention period precludes evaluation of long-term sustainability. Future studies should employ controlled designs (e.g., cluster-randomized or stepped-wedge trials), include more diverse occupational populations, incorporate broader psychosocial outcome measures, and extend the follow-up period to better establish causality and generalizability.

## Conclusion

This 100-day field intervention demonstrated that a structured, incentive-based scientific walking program was associated with significant short-term improvements across a range of chronic disease risk indicators among a sedentary occupational population in Southeast China. The observed reductions in systolic and diastolic blood pressure, body weight, BMI, waist circumference, and body fat percentage highlight the potential of cadence-based brisk walking as a practical and effective non-pharmacological strategy for primary prevention and health promotion. The clear dose–response relationship—where greater adherence to the step goal corresponded to larger health improvements—strengthens the causal inference regarding the intervention’s effectiveness. Furthermore, the high adherence rates achieved through a combination of individual and team-based incentives emphasize the role of motivational support in successful workplace health initiatives. These findings provide meaningful empirical evidence to support the integration of structured physical activity programs into occupational health policies. Future studies employing controlled designs and longer follow-up periods are needed to establish causality and evaluate the long-term sustainability of such health improvements across a wider range of occupational settings.

## Data Availability

The raw data supporting the conclusions of this article will be made available by the authors, without undue reservation.
